# Polyelectrolyte coating of cryo-EM grids improves lateral distribution and prevents aggregation of macromolecules

**DOI:** 10.1107/S2059798322009299

**Published:** 2022-10-20

**Authors:** Dominik Hrebík, Mária Gondová, Lucie Valentová, Tibor Füzik, Antonín Přidal, Jiří Nováček, Pavel Plevka

**Affiliations:** a Central European Institute of Technology, Masaryk University, Kamenice 5, 625 00 Brno, Czech Republic; b Faculty of Agronomy, Mendel University in Brno, Zemědělská 1, 613 00 Brno, Czech Republic; University of Queensland, Australia

**Keywords:** cryo-EM, sample preparation, ssDNA, coating cryo-EM grids, aggregation

## Abstract

Cryo-electron microscopy (cryo-EM) is one of the primary methods used to determine the structures of macromolecules and their complexes. With the increased availability of cryo-electron microscopes, the preparation of high-quality samples has become a bottleneck in the cryo-EM structure-determination pipeline. Macromolecules can be damaged during the purification or prepar­ation of vitrified samples for cryo-EM, making them prone to binding to the grid support, to aggregation or to the adoption of preferential orientations at the air–water interface. Here, it is shown that coating cryo-EM grids with a negatively charged polyelectrolyte, such as single-stranded DNA, before applying the sample reduces the aggregation of macromolecules and improves their distribution. The single-stranded DNA-coated grids enabled the determination of high-resolution structures from samples that aggregated on conventional grids. The polyelectrolyte coating reduces the diffusion of macromolecules and thus may limit the negative effects of the contact of macromolecules with the grid support and blotting paper, as well as of the shear forces on macromolecules during grid blotting. Coating grids with polyelectrolytes can readily be employed in any laboratory dealing with cryo-EM sample preparation, since it is fast, simple, inexpensive and does not require specialized equipment.

## Abstract 2

Coating cryo-EM grids with single-stranded DNA before applying the sample reduces the aggregation of macromolecules and improves their distribution. It enables the determination of structures from samples that aggregate on conventional grids.

## 1. Introduction

Cryo-electron microscopy is the method of choice for the determination of high-resolution structures of biological samples ranging from small proteins to viruses (Cheng, 2018 ▸). As cryo-electron microscopes are becoming common, and the acquisition of electron micrographs is accelerating due to improved automation, sample preparation is becoming a bottleneck in the determination of macromolecular structures (Drulyte *et al.*, 2018 ▸). To collect high-quality cryo-EM data, macromolecules of interest must be vitrified on electron-microscopy grids in their native conformation, separated from each other and in random orientations (Drulyte *et al.*, 2018 ▸). Most often, the grids are prepared by applying 2–4 µl of a sample onto a grid with a holey support and blotting more than 99.9% of the liquid away with filter paper (Drulyte *et al.*, 2018 ▸). Afterwards, the sample is vitrified by rapid plunging into subcooled liquid ethane or a propane–ethane mixture (Drulyte *et al.*, 2018 ▸). However, in many cases macromolecular samples in cryo-EM grids exhibit artifacts such as aggregation, binding to the support or preferential orientations that prevent the acquisition of electron micrographs of sufficient quality to enable high-resolution structure determination (Drulyte *et al.*, 2018 ▸). This undesired behavior of the macromolecules often originates from damage to their structure during the purification or preparation of samples for cryo-EM. Approaches to reducing this undesired sample behavior during the preparation of cryo-EM grids include optimization of the buffer composition (Chari *et al.*, 2015 ▸), the addition of detergents (Chen *et al.*, 2019 ▸), the application of carbon/graphene support layers (Kampjut *et al.*, 2021 ▸; Russo & Passmore, 2014 ▸), the use of affinity grids (Wang, Liu *et al.*, 2020 ▸; Naydenova *et al.*, 2019 ▸; Glaeser & Han, 2019 ▸; Yu *et al.*, 2014 ▸, 2016 ▸; Benjamin *et al.*, 2016 ▸), the use of PEG-based self-assembled monolayers (Meyerson *et al.*, 2014 ▸), PEG-amino coating of graphene oxide grids (Wang, Yu *et al.*, 2020 ▸), PEGylation of macromolecules (Zhang *et al.*, 2021 ▸), the application of a 2D hydrophobin or streptavidin crystal support film (Fan *et al.*, 2021 ▸; Han *et al.*, 2017 ▸) and alternative vitrification methods (Tan & Rubinstein, 2020 ▸; Dandey *et al.*, 2018 ▸; Ravelli *et al.*, 2020 ▸; Koning *et al.*, 2022 ▸; Arnold *et al.*, 2017 ▸). However, extensive optimization of the buffer composition, such as its pH and the concentrations and types of salts and detergents, to prevent undesired sample behavior is tedious and time-consuming because the search depends on a trial-and-error approach. Alternative freezing methods such as Wick-it-off (Tan & Rubinstein, 2020 ▸), Spotiton (Dandey *et al.*, 2018 ▸) and VitroJet (Ravelli *et al.*, 2020 ▸) require the construction or purchase of dedicated instruments.

Here, we show that the application of a polyelectrolyte, such as single-stranded DNA (ssDNA), onto grids prior to the addition of a sample prevents the aggregation of macromolecules and improves their distribution within holes. The modification of the surface with a polyelectrolyte is straightforward: a small droplet of the ssDNA solution is spread over the grid before applying the sample. This approach was tested on three samples that aggregated on ordinary grids. The polyelectrolyte coating of cryo-EM grids is a simple solution that may enable the structure determination of challenging macromolecular samples.

## 2. Materials and methods

### 2.1. Purification of *Tribolium castaneum* hexamerin

*T. castaneum* pupae (5 g) were homogenized in 20 ml lysis buffer (60 m*M* HEPES pH 7.5, 100 m*M* KCl, 10 m*M* MgCl_2_, 4 m*M* DTT, 1% NP-40) using a Dounce homogenizer utilizing pestle A (20 times) followed by pestle B (20 times). The remaining aggregates were disrupted by sonication using a needle-tip sonicator (10 s cycles with amplitude 70, Sonicator Q700, QSonica). The homogenate was centrifuged at 20 000*g* for 10 min at 4°C. A layer of lipidic compounds floated at the top of the supernatant, whereas the pellet contained insoluble cell debris. The clear middle part of the supernatant was saved for further processing. PEG 20 000 was added to the saved protein extract to a final concentration of 8%(*w*/*v*) and incubated on ice for 20 min, followed by centrifugation at 17 500*g* for 10 min at 4°C. The supernatant was discarded and the pellet was centrifuged at 14 500*g* for 1 min to remove the residual PEG in the pellet. The pellet was resuspended in 5 ml buffer (30 m*M* HEPES pH 7.5, 150 m*M* KCl, 5 m*M* MgCl_2_, 2 m*M* DTT) and centrifuged at 20 000*g* for 10 min. The supernatant was treated with TurboNuclease (0.2 µl per 1 ml of sample) for 15 min in a rotary mixer at room temperature and was then centrifuged at 20 000*g* for 10 min. The resulting supernatant was loaded into a Mono Q 5/50 column (GE Healthcare). The column was washed with wash buffer (30 m*M* HEPES pH 7.5) and hexamerin was then gradient eluted with elution buffer (30 m*M* HEPES pH 7.5, 1 *M* KCl). Fractions containing hexamerin were pooled and buffer-exchanged into buffer consisting of 30 m*M* HEPES pH 7.5, 100 m*M* KCl using a PD-10 Desalting column (GE Healthcare). The hexamerin was further purified using size-exclusion chromatography on a Superdex 16/600 200 pg column (GE Healthcare). Fractions containing hexamerin were saved and concentrated to a final concentration of 5 mg ml^−1^ using centrifugal concentrators (100 kDa molecular-weight cutoff; Amicon). The concentration was estimated spectrophoto­metrically using a NanoDrop at a wavelength of 280 nm.

### 2.2. Purification of human 80S ribosome

Adherent HeLa cells were used for the isolation of human 80S ribosomes. HeLa cells were grown in D-MEM (Dulbecco’s Modified Eagle’s Medium, Sigma–Aldrich) supplemented with 10% FBS (fetal bovine serum, Sigma–Aldrich) at 37°C in a 5% CO_2_ environment. For ribosome purification, cells from 200 mm Petri dishes grown to 80% confluency were used. Cells were scraped from the Petri dishes into the medium, centrifuged at 8000*g* for 10 min and transferred into freshly prepared lysis buffer consisting of 25 m*M* HEPES pH 7.5, 15 m*M* MgCl_2_, 300 m*M* NaCl, 0.5%(*v*/*v*) NP-40. The lysate was incubated on ice for 30 min and the cell debris was removed by centrifugation at 10 000*g* for 15 min. To obtain a crude ribosomal pellet, the supernatant was loaded onto a 30% sucrose cushion prepared in buffer consisting of 20 m*M* HEPES pH 7.6, 100 m*M* KCl, 1 m*M* DTT, 10 m*M* NH_4_Cl, 5 m*M* MgCl_2_ and centrifuged overnight at 30 000 rev min^−1^ (105 000*g*) in a Beckman 45Ti rotor (Beckman Coulter). The pellet was resuspended in gradient buffer consisting of 20 m*M* HEPES pH 7.5, 6 m*M* magnesium acetate, 1 m*M* DTT, 0.01%(*w*/*v*) *n*-dodecyl β-d-maltoside (DDM) and then loaded onto a 5–30%(*w*/*w*) linear sucrose-density gradient prepared in gradient buffer using a Gradient Master station (BioComp). The sample was centrifuged for 15 h at 30 000 rev min^−1^ (105 000*g*) using an SW32Ti rotor in a Beckman Optima XPN ultracentrifuge. The gradient was then fractionated from the bottom to the top using a peristaltic pump (Ismatec). The *A*_260_ absorbances of the fractions were measured with a NanoDrop OneC Spectrophotometer (ThermoFisher Scientific) and the peak corresponding to 80S ribosomes was pooled. 80S ribosomes from pooled fractions were pelleted by ultracentrifugation at 50 000 rev min^−1^ (260 000*g*) using a Beckman 70Ti rotor. The final ribosomal pellet was dissolved in buffer composed of 25 m*M* HEPES pH 7.5, 125 m*M* KCl, 5 m*M* magnesium acetate, 1 m*M* DTT and 0.01%(*w*/*v*) DDM. The ribosomes were stored at −80°C until use.

### 2.3. Preparation of ssDNA-coated grids

ssDNA-coated grids were prepared as follows. Commercially obtained salmon-sperm DNA (Sigma–Aldrich, catalogue No. 31149) with an average length of 700 bp was dissolved in nuclease-free water to a final concentration of 10 mg ml^−1^, denatured by heating to 98°C for 10 min and immediately placed on ice. A holey carbon R2/2 300-mesh copper grid (Quantifoil) was treated by glow discharge (Quorum SC7620, 2 s pulse) prior to application of the ssDNA. The grid was then grasped by tweezers and 5 µl of freshly prepared ssDNA was applied onto the carbon side of the grid. After 30 s, the ssDNA solution was blotted with filter paper (Whatman No. 1) from the opposite side (the copper side) of the grid. The grid was immediately used for the standard sample-vitrification procedure with a 20 s waiting time between application of the sample and blotting, as described below for each of the samples.

To exclude the effect of the glow discharge, grid type and operator bias, different glow-discharge devices (Gatan Solaris II and Quorum SC7620), grid types (Quantifoil 2/2, 2/1 and 1.2/1.3) and two different Vitrobot Mark IV devices were used for sample preparation. Moreover, four different operators repeated the ssDNA grid-coating and vitrification procedure. None of the abovementioned modifications had any effect on the reproducibility of the results. One batch of control grids was treated with nuclease-free water without ssDNA and the anti-aggregation effect was not observed.

### 2.4. Cryo-EM sample preparation and 3D reconstruction: *T. castaneum* hexamerin

For vitrification of *T. castaneum* hexamerin, 3.2 µl of hexamerin at a concentration of 0.7 mg ml^−1^ was applied onto a glow-discharged holey carbon grid (Quantifoil R2/2, 300 mesh, copper; control sample) or an ssDNA-coated grid, respectively. The vitrification was performed using an FEI Vitrobot Mark IV (3 s blot time, 0 blot force, 20 s waiting time, 100% humidity at 4°C). Both data sets were collected using a ThermoFisher Titan Krios G2 electron microscope equipped with a K2 summit direct electron detector (Gatan) operating in electron-counting mode using a Bio-quantum energy filter (Gatan) with a slit set to 20 eV. For samples prepared on plain holey carbon grids, the magnification was set to 130 000×, which results in a calibrated pixel size of 1.04 Å; for samples prepared on ssDNA-coated grids the magnification was 165 000× and the corresponding pixel size was 0.822 Å. The defocus range was set from −4.0 to −1.2 µm. Micrographs were acquired with total electron exposures of 63 and 57 e^−^ Å^−2^ for the standard and ssDNA-coated grids, respectively. A total of 1514 and 1711 micrographs were collected from standard and ssDNA-coated grids, respectively.

The mechanical drift and beam-induced movements within the movie frames of one micrograph were corrected with *MotionCor*2 using 5 × 5 patches (Zheng *et al.*, 2017 ▸). The motion-corrected frames were dose-weighted, and defocus values were estimated using *Gctf* (Zhang, 2016 ▸). From the data set collected from plain holey carbon-coated grids, 51 895 hexamerin particles were picked by a combination of manual picking and *crYOLO* (Wagner *et al.*, 2019 ▸). The particles were extracted from the micrographs with a box size of 256 pixels and subjected to reference-free 2D classification in *RELION* 3.1 (Scheres, 2012 ▸), which resulted in the selection of 27 429 particles exhibiting high-resolution features. The particles were re-extracted and recentered with a box size of 256 pixels. The initial 3D reference model was generated by a stochastic gradient-descent algorithm with imposed *D*3 symmetry in *RELION* 3.1. The resulting initial 3D model was used as a starting model for 3D refinement with imposed *D*3 symmetry using the autorefine procedure in *RELION* 3.1. Afterwards, 3D classification with imposed *D*3 symmetry omitting the alignment step was performed, and the class containing the best 10 362 particles was selected. Anisotropic magnification correction and estimation of third- and fourth-order aberration was performed followed by defocus and astigmatism estimation refinement in *RELION* 3.1 (Zivanov *et al.*, 2020 ▸). The particles were then subjected to another round of 3D auto-refinement with imposed *D*3 symmetry, followed by Bayesian polishing using default parameters in *RELION* 3.1 (Zivanov *et al.*, 2019 ▸). The steps of aberration estimation, CTF refinement, Bayesian polishing and auto-refinement were iteratively repeated until no improvement in resolution was achieved. Finally, the post-processing step was performed in *RELION* 3.1, which included threshold masking, *B*-factor sharpening and division by a modulation transfer function. The data set collected on ssDNA-coated grids was 3D reconstructed using essentially the same steps as for the abovementioned sample. The only difference was in the subsequent steps. Initial particle picking by *crYOLO* resulted in 16 616 particles. The 2D classification step selected 8465 particles. The 3D classification selected the final 3106 particles which were used for the final 3D refinement.

### 2.5. Model building and refinement: *T. castaneum* hexamerin

The cryo-electron density map of *T. castaneum* hexamerin was cropped, normalized and assigned space group *P*1. *RaptorX* was used to generate a model of *T. castaneum* hexamerin 2 transcript variant X1 (NCBI Reference Sequence XM_008199125.2; Xu *et al.*, 2021 ▸). The model was manually fitted into the map using *UCSF Chimera* 1.14 (Pettersen *et al.*, 2004 ▸) and rebuilt in *Coot* 0.9.5 (Emsley *et al.*, 2010 ▸). Parts of the structure that were not resolved in the reconstructed map were removed and the loops were remodeled according to the reconstruction. The structure was iteratively refined in real space using *Phenix* 1.19 (Liebschner *et al.*, 2019 ▸) and in recip­rocal space using *REFMAC*5 (Murshudov *et al.*, 2011 ▸), while monitoring the model geometry quality using the *MolProbity* webserver version 4.2 (Chen *et al.*, 2010 ▸). The final refinement was performed in real space using *Phenix* set to enforce *D*3 noncrystallographic symmetry constraints.

### 2.6. Cryo-EM sample preparation and 3D reconstruction: human 80S ribosome

For vitrification of human 80S ribosome, 3.0 µl of ribosome sample at a concentration of 1.2 mg ml^−1^ was applied onto a glow-discharged holey carbon grid (Quantifoil R2/2, 300 mesh, copper; control sample) or an ssDNA-coated grid. The vitrification was performed using an FEI Vitrobot Mark IV (3 s blot time, 0 blot force, 30 s waiting time, 100% humidity at 4°C). The data set from the ssDNA-coated grid was collected on a ThermoFisher Titan Krios G2 electron microscope equipped with a Falcon 3 direct electron detector (ThermoFisher Scientific) running in integrating mode. The magnification was set to 75 000×, which resulted in a calibrated pixel size of 1.063 Å. The defocus range was set from −2.4 to −0.6 µm. Micrographs were acquired with a total electron exposure of 64 e^−^ Å^−2^. A total of 4527 micrographs were collected. The 3D reconstruction workflow was the same as that for *T. castaneum* hexamerin, with the following modifications. The box size used for the extraction of particles was 512 pixels, and 133 524 particles were automatically picked by *crYOLO* and subsequently extracted by *RELION* 3.1. After the reference-free 2D classification 124 986 particles were retained. Using the 3D classification, we selected 66 372 particles which were used in the final 3D auto-refinement, with the *side-splitter* algorithm used as the external reconstruction script in *RELION* 3.1 (Ramlaul *et al.*, 2020 ▸).

### 2.7. Model refinement: human 80S ribosome

The structure of human 80S ribosome (PDB entry 6qzp; Natchiar *et al.*, 2017 ▸) was used as a starting model. The structure was fitted into the cryo-EM map using *ChimeraX* 1.2.5 (Pettersen *et al.*, 2021 ▸). Real-space refinement was performed using *Phenix* 1.20, with the starting model used as a reference restraint. Visual inspection of the model and cross-correlation validation was performed in *Coot* 0.9 and *ChimeraX* 1.2.5 (Emsley *et al.*, 2010 ▸; Pettersen *et al.*, 2021 ▸). Final geometry evaluation of the model was performed using the *MolProbity* webserver version 4.2 (Chen *et al.*, 2010 ▸).

### 2.8. Acquisition and reconstruction of tomograms of human 80S ribosome

Tilt series of human ribosome samples were collected on grids vitrified under the same conditions as the grids used for single-particle data collection. Data sets for both ribosomes vitrified on a standard holey carbon grid and ribosomes vitrified on an ssDNA-coated grid were collected using a ThermoFisher Arctica electron microscope equipped with a Falcon 3 direct electron detector (ThermoFisher Scientific) in integrating mode. Tilt series were collected using *SerialEM* (Schorb *et al.*, 2019 ▸), with a tilt range of −56° to +56° and a step increment of 2°, using the dose-symmetric acquisition scheme (Turoňová *et al.*, 2020 ▸). The tilt series were collected with a total exposure of 180 e^−^ Å^−2^ and a defocus of −6 µm. The magnification was set to 28 000×, which resulted in a pixel size of 5.19 Å. Acquired tilt series were aligned in *IMOD* 4.11 (Kremer *et al.*, 1996 ▸) by cross-correlation between subsequent tilts. Tomogram positioning was performed manually. The tomograms were reconstructed using a weighted back-projection algorithm. Tomograms were visualized using 3*dmod* (Kremer *et al.*, 1996 ▸).

### 2.9. Cryo-EM sample preparation and 3D reconstruction: apoferritin

The apoferritin sample was kindly provided by ThermoFisher Scientific in Brno. For vitrification, 3.1 µl of apoferritin at a concentration of 4.0 mg ml^−1^ in 30 m*M* HEPES pH 7.5, 150 m*M* NaCl, 1 m*M* DTT buffer was applied onto a standard or ssDNA-coated holey carbon grid (Quantifoil R2/2, 300 mesh, copper). The vitrification was performed using an FEI Vitrobot Mark IV (6 s blot time, 30 s waiting time, 0 blot force, 100% humidity at 4°C). The data set was collected from the ssDNA-coated holey carbon grid using a ThermoFisher Titan Krios G4 electron microscope in the ThermoFisher Scientific factory in Brno equipped with a Falcon 4 direct electron detector (ThermoFisher Scientific) running in electron-counting mode installed behind a Selectris energy filter with a slit set to 10 eV. The magnification was set to 120 000×, which resulted in a calibrated pixel size of 0.4525 Å. The defocus range was −0.3 to −1.7 µm. Micrographs were acquired with a total electron exposure of 40 e^−^ Å^−2^. A total of 3282 micrographs were collected. The beam-induced movements within one micrograph were corrected with the *RELION* 4.0-beta implementation of *MotionCor*2 using 5 × 5 patches. The motion-corrected micrographs were dose-weighted, and defocus values were estimated using *CTFFIND*4 (Rohou & Grigorieff, 2015 ▸). Particles were automatically picked by *crYOLO* and subsequently extracted using *RELION* 4.0-beta (Kimanius *et al.*, 2021 ▸) using a box size of 512 pixels. A total of 206 030 particles were extracted. Particles were 4× binned before the initial 2D classification. After two rounds of reference-free 2D classification, 162 299 particles were selected for further processing. A map of apoferritin (EMDB entry EMD-24665; Zhang *et al.*, 2020 ▸) low-pass filtered to 30 Å was used as an initial model for 3D auto-refinement with imposed octahedral symmetry in *RELION* 4.0-beta. Subsequently, 3D classification with imposed octahedral symmetry omitting the alignment step was performed in *RELION* 4.0-beta, which selected 156 560 particles for further processing. These particles were re-extracted with a box size of 512 pixels. Another round of 3D auto-refinement with imposed octahedral symmetry was performed, followed by 3D classification with imposed octahedral symmetry omitting the alignment step, which yielded the final set of 65 786 particles. These particles were subjected to magnification correction and the estimation of third- and fourth-order Zernike polynomials in *RELION* 4.0-beta (Zivanov *et al.*, 2020 ▸). The aberration-corrected particles were further subjected to per-particle defocus and per-micrograph astigmatism and CTF envelope function estimation (CTF *B*-factor fitting). Ewald sphere-corrected 3D reconstruction was performed as implemented in *relion_reconstruct* in *RELION* 4.0-beta (Russo & Henderson, 2018 ▸). The particles were subjected to another round of 3D auto-refinement with imposed octahedral symmetry followed by Bayesian polishing with default parameters in *RELION* 4.0-beta (Zivanov *et al.*, 2019 ▸). Another round of CTF refinement was repeated for magnification anisotropy, optical aberration, per-particle defocus and per-micrograph astigmatism estimation. The CTF refinement was followed by 3D auto-refinement with imposed octahedral symmetry and another round of Bayesian polishing. Finally, 3D auto-refinement followed by Ewald sphere correction was performed. The post-processing step, which included threshold masking and *B*-factor sharpening, was performed in *RELION* 4.0-beta.

### 2.10. Model building and refinement: apoferritin

The structure of apoferritin (PDB entry 7k3w) was used as a starting model for structure determination (Zhang *et al.*, 2020 ▸). The model was fitted to the cryo-EM map using *ChimeraX* 1.2.5 (Pettersen *et al.*, 2021 ▸). Real-space refinement was then performed in *Phenix* 1.20 with the starting model used as a reference restraint (Liebschner *et al.*, 2019 ▸). Visual inspection of the model and validation was performed in *Coot* 0.9 and *ChimeraX* (Emsley *et al.*, 2010 ▸; Pettersen *et al.*, 2021 ▸). Final geometry evaluation of the model was performed using the *MolProbity* webserver version 4.2 (Chen *et al.*, 2010 ▸).

### 2.11. Testing of polymers for grid coating

The following polymers were tested as grid coatings: polyglutamic acid with an average molecular weight of 50 000–100 000 Da (Sigma–Aldrich), poly(sodium 4-styrenesulfonate) with an average molecular weight of 200 000 Da (Sigma–Aldrich), polyethylene glycol with an average molecular weight of 35 000 Da (Sigma–Aldrich) and poly(l-lysine) hydrobromide (Sigma–Aldrich) with an average molecular weight of 70 000–150 000 Da. The polyelectrolytes were diluted to a final concentration of 10 mg ml^−1^ in nuclease-free water and were used in the preparation of polymer-coated grids with the same protocol as that used for the ssDNA-coated grids.

## 3. Results and discussion

### 3.1. Coating cryo-EM grids with polyelectrolytes prior to sample application

**Figure 1:**
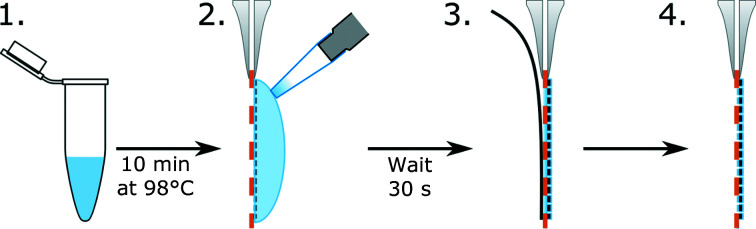
Preparation of ssDNA-coated cryo-EM grids. (1) Before application onto the cryo-EM holey carbon grid, dsDNA is heated to 98°C for 10 min to obtain ssDNA. The ssDNA solution is then immediately placed on ice to prevent the formation of double-stranded DNA. (2) 5 µl of the ssDNA is applied onto a holey grid. (3) After 30 s, the ssDNA is blotted from the opposite side of the grid. (4) The ssDNA-coated grid is ready to be used for a regular sample-vitrification procedure.

**Figure 2:**
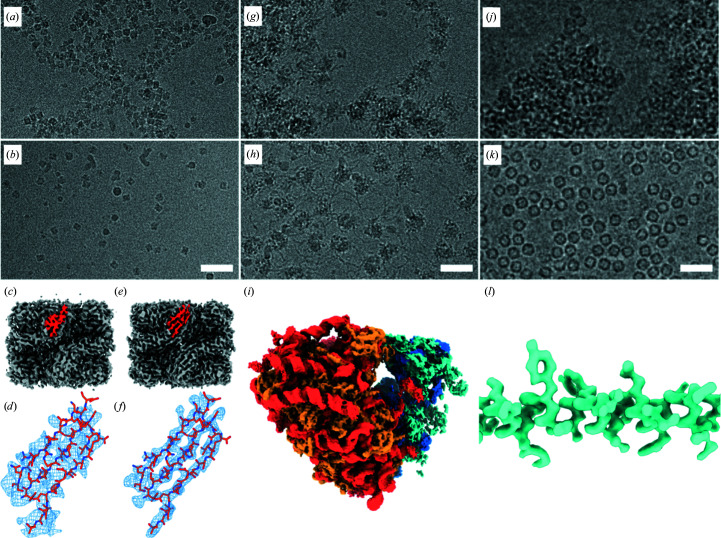
Comparison of samples vitrified on regular holey carbon grids and ssDNA-coated grids. Samples of hexamerin (*a*–*f*), human 80S ribosome (*g*–*i*) and apoferritin (*j*–*l*) vitrified on regular holey carbon grids (*a*, *g*, *j*) and ssDNA-coated holey carbon grids (*b*, *h*, *k*). The samples on regular grids (*a*, *g*, *j*) are aggregated, whereas those on ssDNA-coated grids (*b*, *h*, *k*) are monodisperse. The white scale bars in the micrographs represent 30 nm. (*c*, *d*) 3D cryo-EM reconstructions of hexamerin calculated from images obtained from a regular holey carbon grid (*c*) with highlighted detail (red β-sheet) from the map (*d*). (*e*, *f*) 3D cryo-EM reconstructions of hexamerin obtained from an ssDNA-coated cryo-EM grid. (*i*, *l*) Cryo-EM reconstructions of apoferritin (*i*) and human 80S ribosome (*l*) obtained from samples vitrified on ssDNA-coated grids.

The coating of grids with polyelectrolytes prior to sample application was tested using the following procedure. (i) A solution of a polyelectrolyte with a concentration of 10 mg ml^−1^ in nuclease-free water was heated to 98°C for 10 min (Fig. 1 ▸). (ii) 5 µl of the polyelectrolyte solution was pipetted onto a plasma-treated holey carbon grid and incubated for 30 s (Fig. 1 ▸). (iii) The excess solution was blotted from the other side of the grid (Fig. 1 ▸). (iv) The polyelectrolyte-coated grid was used for standard plunge-freezing sample preparation (Fig. 1 ▸). The tested polyelectrolytes included DNA, polyglutamic acid, poly(sodium 4-styrene­sulfonate) (PSS), polyethylene glycol (PEG) and poly(l-lysine) (pLys). The resulting grids were imaged using transmission cryo-electron microscopy. To eliminate the effects of grid-to-grid variability, which is high in the preparation of samples for cryo-EM, we tested all of the electrolytes using at least three independently prepared grids. The application of negatively charged polyelectrolytes to the grids prior to the sample eliminated aggregation and improved the distribution of particles in the vitreous ice (Fig. 2 ▸, Supplementary Fig. S1).

### 3.2. Test sample 1: hexamerin from *T. castaneum*

Hexamerin is a 57 kDa protein from the haemolymph of insect larvae and pupae (Burmester, 1999 ▸). It forms homohexamers with *D*3 symmetry. Purified *T. castaneum* hexamerin at a concentration of 5 mg ml^−1^ in pH 7.5 buffer containing 30 m*M* HEPES and 100 m*M* KCl was used in the experiments. When vitrified on standard holey carbon grids, *T. castaneum* hexamerin aggregated, rendering most of the particle images useless for single-particle reconstruction (Fig. 2 ▸ *a*, Supplementary Fig. S1*a* ). The hexamerin structure was determined to an overall resolution of 3.1 Å (Fig. 2 ▸ *d*, Supplementary Fig. S2*a* , Supplementary Table S1). The surface parts of the complex were poorly resolved, which prevented building of their structure (Figs. 2 ▸ *c* and 2 ▸ *d*). In contrast, the same sample of hexamerin vitrified on DNA-coated grids exhibited no particle aggregation (Fig. 2 ▸ *b*, Supplementary Fig. S1*b* ). The auto-picking procedure, as implemented in *crYOLO*, efficiently identified the hexamerin particles (Wagner *et al.*, 2019 ▸). The final structure reached a resolution of 3.1 Å (Figs. 2 ▸ *e* and 2 ▸ *f*, Supplementary Fig. S2*b* ) and the surface regions of the cryo-EM map of the hexamer were of sufficient quality to enable model building (Figs. 2 ▸ *e* and 2 ▸ *f*).

### 3.3. Test sample 2: human 80S ribosome

Human 80S ribosome has a molecular weight of 4.3 MDa (Khatter *et al.*, 2015 ▸). The first cryo-EM structure of human 80S ribosome was solved to a resolution of 3.6 Å by Bruno Klaholz’s group in 2015 (Khatter *et al.*, 2015 ▸). The test sample of 80S ribosome at a concentration of 1.2 mg ml^−1^ in 25 m*M* HEPES pH 7.5, 125 m*M* KCl, 5 m*M* magnesium acetate, 1 m*M* DTT, 0.01% DDM buffer exhibited nearly complete particle aggregation on standard cryo-EM grids (Fig. 2 ▸ *g*, Supplementary Fig. S1*c* ). Electron micrographs from these grids could not be used for cryo-EM reconstruction. Ordinarily, samples of human 80S ribosome are well behaved, as demonstrated by numerous high-resolution structures. The sample used in our experiments was damaged during purification and therefore exhibited aggregation during sample preparation using standard cryo-EM grids. In contrast, DNA-coated grids contained separated particles (Fig. 2 ▸ *h*, Supplementary Fig. S1*d* ). The final reconstruction from 22 000 particles achieved a resolution of 3.8 Å (Fig. 2 ▸ *i*, Supplementary Fig. S2*c* , Supplementary Table S1). The coating of grids with DNA was crucial for the structure determination of the 80S ribosome from our sample. The structure solved from the DNA-coated grids is identical to the previously solved ribosome structure, with a cross-correlation of 0.92 between the two cryo-EM maps.

### 3.4. Test sample 3: human apoferritin

Apoferritin is one of the most popular samples for benchmarking cryo-EM methods because of its stability and 24-fold cubic symmetry (Wu *et al.*, 2020 ▸). Reconstructions of apoferritin routinely achieve resolutions of better than 2 Å and the highest resolution approached 1.2 Å (Wu *et al.*, 2020 ▸; Nakane *et al.*, 2020 ▸; Yip *et al.*, 2020 ▸; Zhang *et al.*, 2020 ▸). Our test sample of apoferritin exhibited extensive aggregation on standard holey carbon grids, which prevented the use of the images for single-particle reconstruction (Fig. 2 ▸ *j*, Supplementary Fig. S1*e* ). The aggregation of apoferritin seen in our experiments was probably caused by damage to the sample during purification, as discussed below. In contrast, ssDNA-coated grids enabled the recording of data that could be used to reconstruct its structure to a resolution of 1.77 Å (Fig. 2 ▸ *l*, Supplementary Figs. S1*f* and S2*d* , Supplementary Table S1).

### 3.5. Grids coated with ssDNA enable high-resolution structure determination

**Figure 3:**
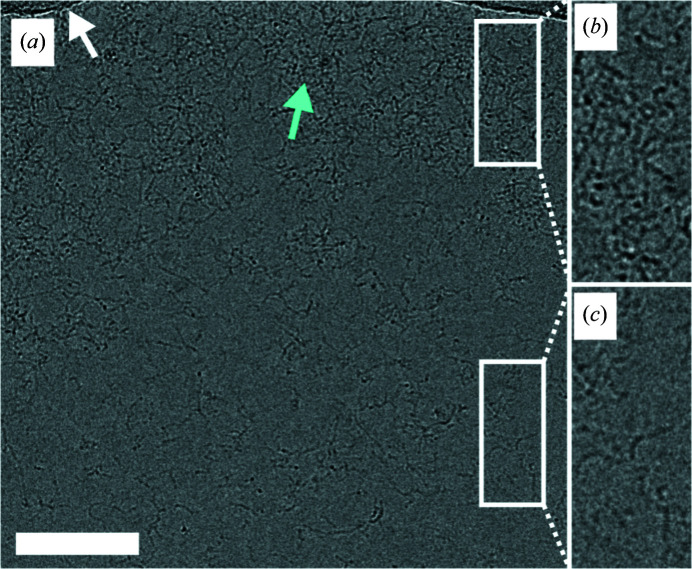
Distribution of ssDNA in grid holes upon vitrification. (*a*) Transmission electron micrograph of an ssDNA-coated grid vitrified without macro­molecular sample. The carbon edge and entangled strands of ssDNA are indicated with white and cyan arrows, respectively. (*b*, *c*) The insets show details of the entangled ssDNA strands at different distances from the carbon edge. The white scale bar represents 100 nm.

Electron micrographs of ssDNA-coated grids vitrified without a macromolecular sample show strands of DNA extending from the edges towards the center of the holes (Fig. 3 ▸). The DNA strands behave the same way in grids with macromolecular samples; however, the DNA is less visible, probably due to thicker ice (Fig. 1 ▸). The DNA strands increase the background around the macromolecules of interest. Nevertheless, the presence of DNA on the grids did not prevent the reconstructions from achieving high resolution; for apo­ferritin, this was 1.77 Å (Fig. 2 ▸ *l*, Supplementary Fig. S2*d* , Supplementary Table S1).

### 3.6. Properties of polyelectrolytes that are required to prevent sample aggregation

**Figure 4:**
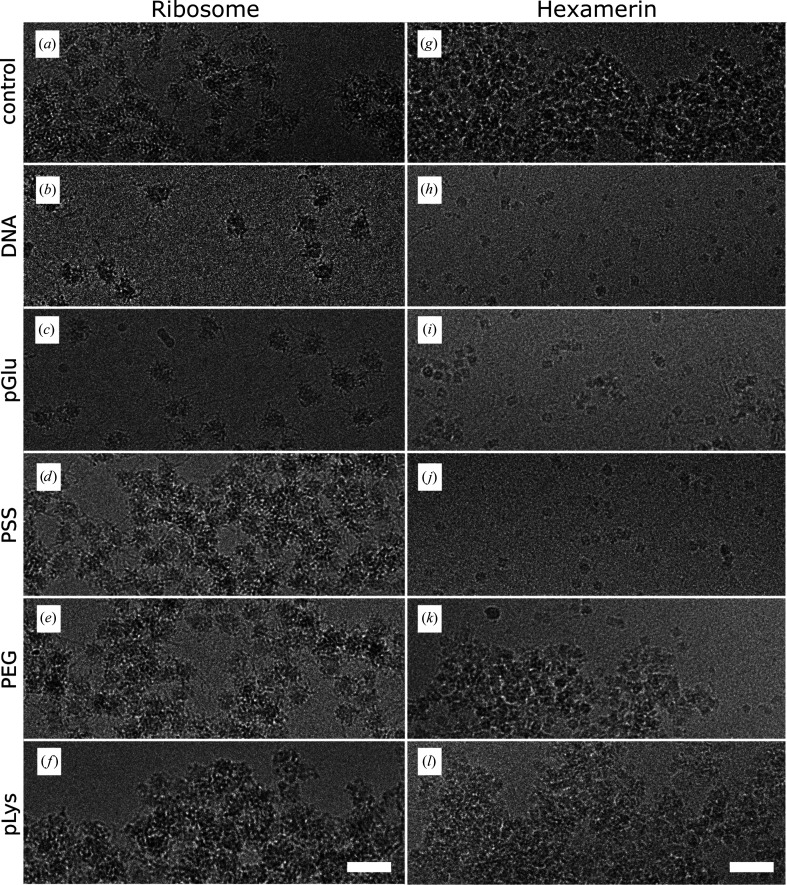
Effect of polyelectrolytes with various properties on sample aggregation. (*a*, *g*) Control samples of human 80S ribosome (*a*) and hexamerin (*g*) vitrified on regular holey carbon grids. (*b*–*f*, *h*–*l*) Samples of human 80S ribosome (*b*–*f*) and hexamerin (*h*–*l*) vitrified on grids coated with various polyelectrolytes. (*b*–*d*, *h*–*j*) The negatively charged polyelectrolytes ssDNA (*b*, *h*), pGlu (*c*, *i*) and PSS (*d*, *j*). (*e*, *k*) PEG was tested as a polyelectrolyte with no overall charge. (*f*, *l*) pLys was tested as a positively charged polyelectrolyte. The white scale bars in the micrographs represent 30 nm.

Polyelectrolytes with positive, neutral and negative charges were tested to determine whether the anti-aggregation effect of the grid coating on macromolecule behavior is charge-dependent. The length of the polymers was chosen to match that of the ssDNA segments used in the initial experiments. We tested negatively charged polyglutamic acid (pGlu) with molecular weight 50 000–100 000 Da and poly(sodium 4-styrenesulfonate) (PSS) with an average molecular weight of 200 000 Da, neutral polyethylene glycol (PEG) with an average molecular weight of 35 000 Da and positively charged poly(l-lysine) (pLys) with a molecular weight of 70 000–150 000 Da. Grids pre-coated with pGlu and PSS improved the sample quality relative to the noncoated grids, but to a lesser extent than those coated with ssDNA (Figs. 4 ▸ *b*–4 ▸ *d* and 4 ▸ *h*–4 ▸ *j*). PSS had no anti-aggregation effect on the 80S ribosome sample (Fig. 4 ▸ *d*). It has been shown that PSS has only a minimal effect in increasing the solution viscosity (Tian *et al.*, 2015 ▸). In contrast, addition of ssDNA and pLys to a solution increases its viscosity (Laesecke & Burger, 2014 ▸; Yaron & Berger, 1963 ▸). Therefore, the local increase in sample viscosity at a grid surface may contribute to the anti-aggregation effect of the polyelectrolyte grid coating. The behavior of samples vitrified on PEG-coated grids did not differ from those on control grids (Fig. 4 ▸ *e* and 4 ▸ *k*), and the coating of grids with pLys exacerbated sample aggregation (Figs. 4 ▸ *f* and 4 ▸ *l*). In combination, the results provide evidence that a negative charge of the grid-coating compound is essential for the anti-aggregation effect on the tested macromolecules. Hexamerin, human 80S ribosome and apoferritin are soluble macromolecular complexes with a predominantly negative surface charge (Supplementary Fig. S3). Therefore, coating grids with ssDNA has the potential to improve the quality of most macromolecular samples. We speculate that macromolecules or complexes with overall positive charge, such as histones, may benefit from coating grids with a positively charged polymer such as pLys.

### 3.7. ssDNA coating enables the preparation of useful grids from suboptimal macromolecular samples

A common method for preparing cryo-EM grids is to blot away the excess sample with filter paper prior to vitrification (Dubochet *et al.*, 1988 ▸). It has been speculated that macromolecules may be damaged during sample blotting by exposure to the shearing forces of the flowing liquid or by interactions with the carbon support, filter paper or air–water interface (Chen *et al.*, 2019 ▸; Glaeser, 2018 ▸; Glaeser & Han, 2017 ▸; Armstrong *et al.*, 2020 ▸; Lyumkis, 2019 ▸; D’Imprima *et al.*, 2019 ▸; Noble *et al.*, 2018 ▸; Zheng *et al.*, 2000 ▸). The samples of hexamerin, human 80S ribosome and apoferritin used in our experiments aggregated on standard cryo-EM grids but not on ssDNA-coated grids, providing evidence that the aggregation occurred during the blotting procedure on standard cryo-EM grids. When control grids were treated with nuclease-free water without ssDNA, the anti-aggregation effect was not observed.

The mean shortest distances between automatically boxed particles of hexamerin, which has a diameter of 13 nm, in electron micrographs recorded on standard and ssDNA-coated grids were 16 ± 2 and 40 ± 13 nm, respectively. For the human 80S ribosome, which has a diameter of 25 nm, the distances were 29 ± 4 and 39 ± 9 nm, respectively. It was not possible to calculate the distances between the particles of apoferritin on standard grids because the automated boxing procedure failed due to extreme sample aggregation. The mean nearest-neighbor distances of hexamerin and 80S ribosomes on standard grids are close to the respective particle diameters, indicating that the samples were aggregated. In contrast, on the ssDNA-coated grids the particles were sufficiently separated.

**Figure 5:**
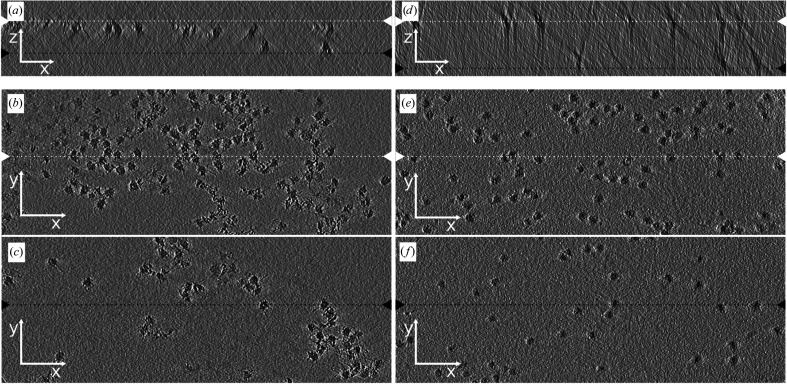
Tomograms of human 80S ribosome vitrified on a regular holey carbon grid and an ssDNA-coated grid. Samples of human 80S ribosome vitrified on regular (*a*–*c*) and ssDNA-coated (*d*–*f*) grids. (*a*, *d*) *XZ* planes from tomograms with the upper and lower air–water interfaces indicated by white and black triangles with dotted lines of the same colors, respectively. (*b*, *e*) *XY* planes from the top sections of the tomograms. (*c*, *f*) *XY* planes from the bottom sections of the tomograms. The white and black triangles and dotted lines in (*b*)–(*f*) indicated the positions of the cross-sections shown in (*a*) and (*d*).

Cryo-tomograms of human 80S ribosomes vitrified on regular holey carbon grids and ssDNA-coated grids show that ribosomes accumulated at the air–water interface on both types of grids (Fig. 5 ▸). This demonstrates that the polyelectrolyte coating does not prevent the macromolecules from reaching the air–water interface, which probably does not play an important role in inducing the aggregation of macromolecules. It should be noted that well prepared samples of apoferritin and 80S human ribosome do not aggregate on standard cryo-EM grids (Khatter *et al.*, 2015 ▸; Wu *et al.*, 2020 ▸), indicating that the macromolecular complexes used in our experiments were damaged during purification or improper sample storage. Therefore, the ssDNA coating of grids enables the preparation of useful samples for cryo-EM, even from macromolecular samples that had undergone a suboptimal purification procedure. The ssDNA coating of grids improved the sample distribution in holey carbon holes regardless of the thickness of the vitreous ice, indicating that the positive effect of polyelectrolyte coating is not due to increasing the ice thickness. We speculate that the positive effects of the polyelectrolyte coating of grids stem from the ability of the coating to charge-shield the grid surface and macromolecules and to limit the diffusion of macromolecules and thus reduce the effects of shear forces by a local increase in viscosity and decrease the frequency of macromolecular contact with the grid support and blotting paper.

## 4. Conclusions

Here, we show that coating the cryo-EM grid surface with ssDNA, or other negatively charged polyelectrolytes, reduced sample aggregation and improved the distribution of macromolecules in holey carbon grids for cryo-EM. The samples used for testing, hexamerin, human 80S ribosome and apoferritin, exhibited undesirable properties such as protein aggregation and/or affinity for the carbon support on standard cryo-EM grids. The use of polyelectrolyte-coated grids was essential for the determination of macromolecular structures from these samples. We also showed that the background due to the presence of ssDNA on the grids does not prevent the reconstructions from achieving high resolution; for apo­ferritin, this was 1.77 Å (Fig. 2 ▸ *l*, Supplementary Fig. S2*d* , Supplementary Table S1). The anti-aggregation effect was dependent on the negative charge of the polyelectrolyte used for the grid coating; neutral or positively charged poly­electrolytes decreased the sample quality (Figs. 4 ▸ *e*, 4 ▸ *f*, 3 ▸ *k* and 4 ▸ *l*). The coating of grids with ssDNA is simple, does not require any additional specialized equipment or chemicals and can be performed immediately prior to the preparation of grids for cryo-EM.

## Supplementary Material

EMDB reference: hexamerin on ssDNA-coated grid, EMD-14679

EMDB reference: hexamerin on holey carbon grid, EMD-14680

EMDB reference: human 80S ribosome on ssDNA-coated grid, EMD-14704

EMDB reference: apoferritin on ssDNA-coated grid, EMD-14705

PDB reference: hexamerin on ssDNA-coated grid, 7ze1

PDB reference: apoferritin on ssDNA-coated grid, 7zg7

PDB reference: human 80S ribosome on ssDNA-coated grid, 7zfw
